# Major vault protein in cardiac and smooth muscle

**Published:** 2016-05-23

**Authors:** Nataliia V. Shults, Dividutta Das, Yuichiro J. Suzuki

**Affiliations:** Department of Pharmacology and Physiology, Georgetown University Medical Center, Washington, DC 20057, USA

**Keywords:** cardiac muscle, heart, lung, major vault protein, vault, smooth muscle

## Abstract

Major vault protein (MVP) is the major component of the vault particle whose functions are not well understood. One proposed function of the vault is to serve as a mechanism of drug transport, which confers drug resistance in cancer cells. We show that MVP can be found in cardiac and smooth muscle. In human airway smooth muscle cells, knocking down MVP was found to cause cell death, suggesting that MVP serves as a cell survival factor. Further, our laboratory found that MVP is S-glutathionylated in response to ligand/receptor-mediated cell signaling. The S-glutathionylation of MVP appears to regulate protein-protein interactions between MVP and a protein called myosin heavy chain 9 (MYH9). Through MYH9 and Vsp34, MVP may form a complex with Beclin-1 that regulates autophagic cell death. In pulmonary vascular smooth muscle, proteasome inhibition promotes the ubiquitination of MVP, which may function as a mechanism of proteasome inhibition-mediated cell death. Investigating the functions and the regulatory mechanisms of MVP and vault particles is an exciting new area of research in cardiovascular/pulmonary pathophysiology.

## Introduction to vault and major vault protein (MVP)

In 1986, Kedersha and Rome^[5]^ reported their discovery of a novel ribonucleoprotein particle in rat liver-coated vesicle preparations with a barrel-like structure resembling the multiple arches forming cathedral vaults. The authors named these particles ‘vaults’, which were found to be largely composed of a protein with a relative molecular mass of 104,000 subsequently named MVP.

Later, Scheper *et al*.^[14]^ reported the discovery of a protein with a relative molecular mass of 110,000 that is overexpressed in P-glycoprotein (Mdr1)-negative multidrug-resistant tumor cell lines. This protein was named ‘lung-related resistance protein’. The cloning of this gene revealed that lung-related resistance protein is MVP^[12]^.

The findings that MVP^[14]^ and vaults^[6]^ are upregulated in multidrug-resistant cancer cells supported the concept that vaults play a role in drug resistance. MVP has been proposed to export drugs from the nucleus for sequestration in cytosolic vesicles^[1, 9]^ and regulate drug resistance in concert with ABC transporters^[13]^. In drug-resistant and MVP-overexpressing non-small cell lung cancer cells, MVP was co-localized with doxorubicin in cytoplasmic vesicles^[10]^. The siRNA knockdown of MVP inhibited the sequestration of doxorubicin and promoted drug accumulation and cytotoxicity^[4]^.

Contrary to the ample evidence for the role of MVP in multidrug resistance in cancer cells, some reports do not support this hypothesis. First, Siva *et al*.^[15]^ reported that the upregulation of vaults was not sufficient to induce multidrug resistance. These findings can be explained, however, by the concept that the post-translational activation of MVP may be needed. Second, MVP knockout and thus vault-less mice were reported not to be hypersensitive to neoplastic drugs^[11]^. These experiments, however, were performed in non-cancerous cells and did not account for the activated MVP state, which might occur in cancer cells.

While the function and regulation of MVP remain unclear, the structure of vaults has been well understood. In addition to MVP, two minor proteins form the vault, namely 193-kDa vault poly(ADP-ribose) polymerase (vPARP)^[7]^ and 240-kDa telomerase-associated protein-1 (TEP1)^[8]^. The vault also contains four RNAs with sizes of 88, 88, 98 and 101 bases. Recently, the structure of the rat liver vault was obtained at 3.5-angstrom resolution, which revealed that a vault comprises of 78 MVP chains^[16]^.

## MVP is expressed in smooth muscle and cardiac muscle

**Figure 1:**
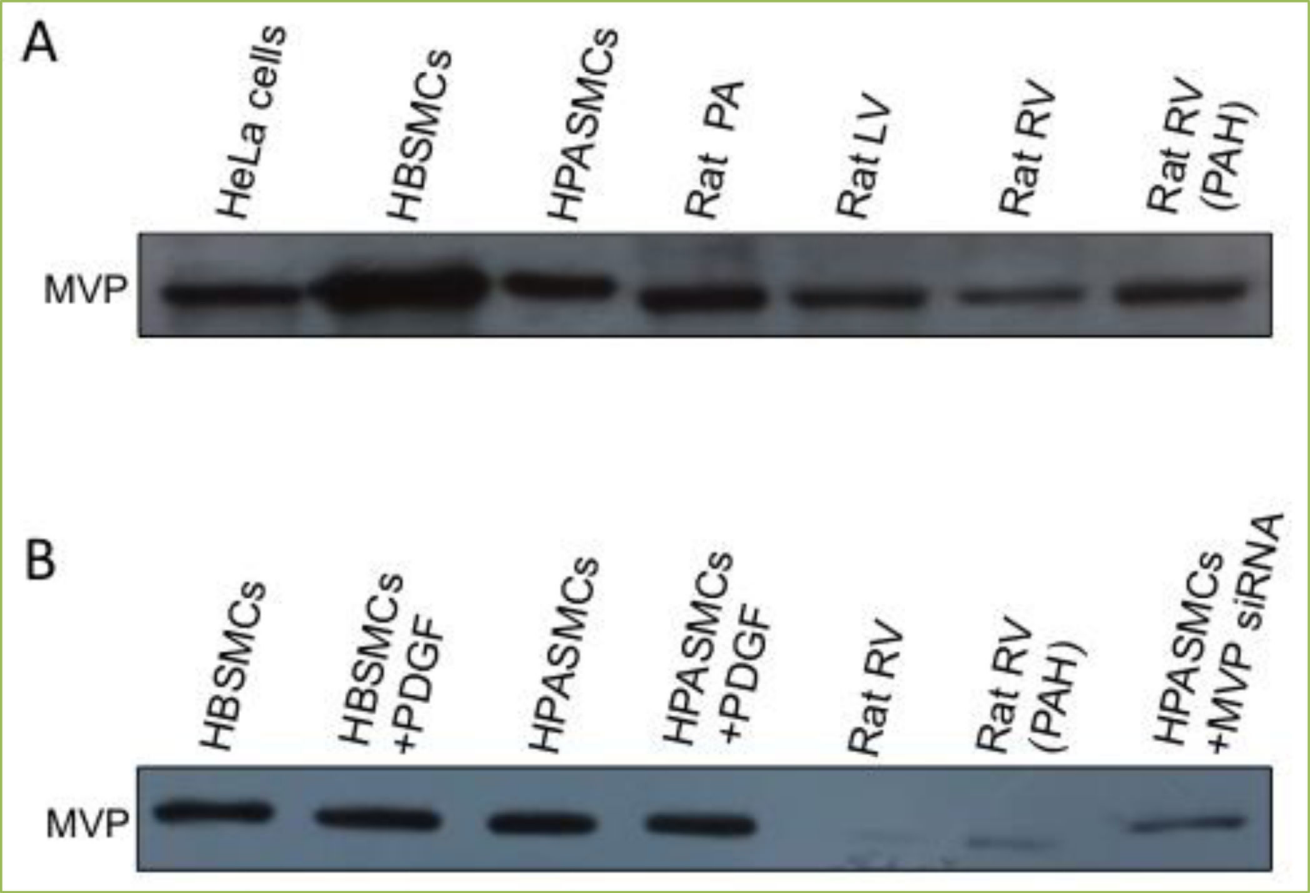
Western blotting shows that cardiac and smooth muscle express MVP Cell lysates or tissue homogenates of (A) HeLa human cervical cancer cells, human bronchial smooth muscle cells (HBSMCs), human pulmonary artery smooth muscle cells (HPASMCs), isolated rat pulmonary artery (PA), rat left ventricle (LV), rat right ventricle (RV) and the RV from a rat with pulmonary arterial hypertension (PAH) and (B) untreated HBSMCs, HBSMCs treated with platelet-derived growth factor (PDGF) for 24 h, untreated HPASMCs, HPASMCs treated with PDGF, normal rat RV, the RV from a rat with PAH and HPASMCs in which MVP treated with MVP siRNA were subjected to Western blotting to monitor MVP levels. MVP siRNA and antibody were purchased from Santa Cruz Biotechology (Dallas, TX, USA).

**Figure 2:**
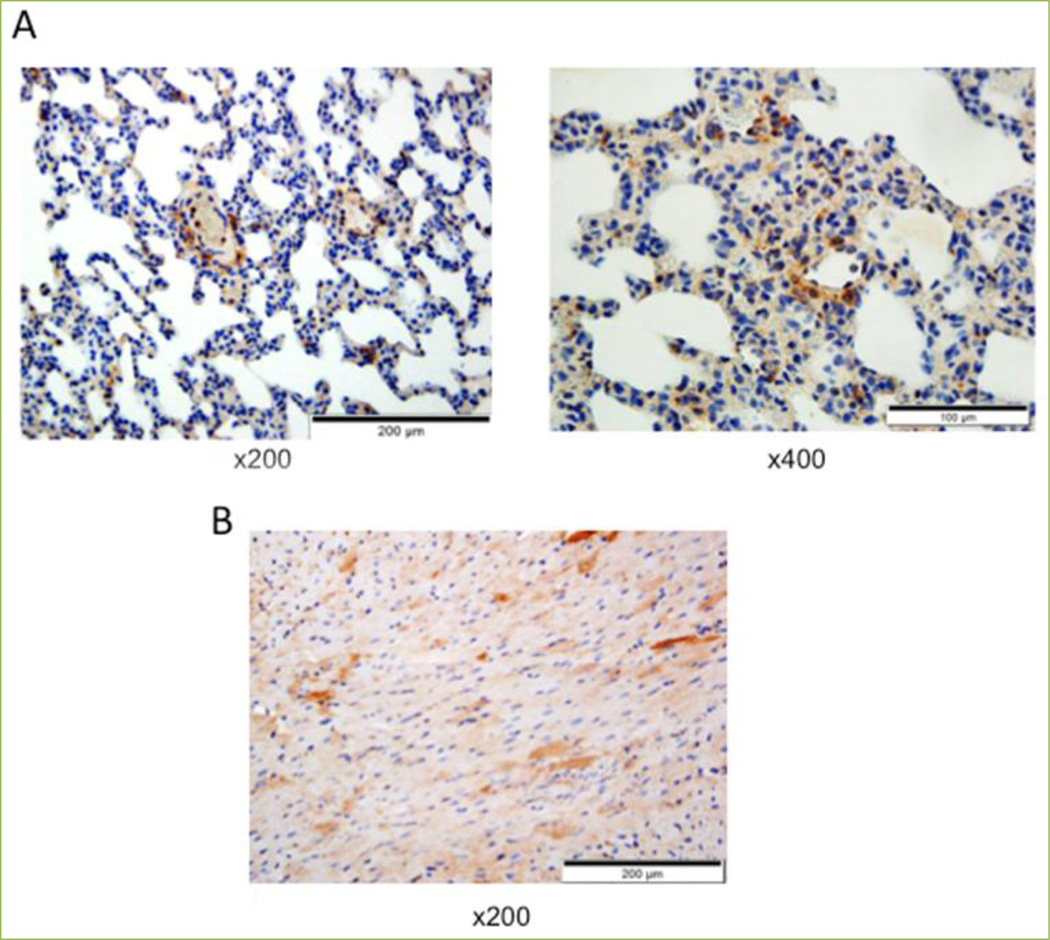
Immunohistochemistry shows that cardiac and smooth muscle express MVP Rat lung (A) and heart (B) tissues were immersed in buffered 10% formalin at room temperature and embedded in paraffin. Paraffin-embedded tissues were cut and mounted on glass slides. Tissue sections were subjected to immunohistochemistry with the MVP antibody (Santa Cruz).

To our knowledge, studies of MVP in smooth muscle cells (SMCs) had never been conducted until our publications, which show that human airway SMCs^[2]^ and lung vascular SMCs^[17]^ express MVP. Fig. 1A shows that MVP is expressed in the primary culture of human bronchial smooth muscle cells (HBSMCs), human pulmonary artery smooth muscle cells (HPASMCs) and isolated rat pulmonary artery (PA) as monitored by Western blotting. The human smooth muscle cell MVPs migrated in a similar way to MVP in HeLa human cervical carcinoma cells. As human MVP contains 893 amino acids and rat MVP contains 861 amino acids, smaller rat MVP migrated a little more in the SDS polyacrylamide gel than the human MVP molecule. MVP was also detected in both growth-arrested and proliferating smooth muscle cells in the presence of platelet-derived growth factor (PDGF). The siRNA knockdown of MVP in HPASMCs validates that the band observed is indeed MVP (Fig. 1B). Immunohistochemistry also demonstrates the expression of MVP in the pulmonary vascular smooth muscle, which was remarkably found to be higher than that in any other parts of the lung in normal healthy rats (Fig. 2A).

The expression of MVP was also found in the heart. Fig. 1 shows that MVP is expressed in the left ventricle (LV) and the right ventricle (RV) of the heart in normal rats as well as in the hypertrophied RV in rats with pulmonary arterial hypertension (PAH). Immunohistochemistry also shows the expression of MVP in cardiac muscle as well as in coronary artery smooth muscle in normal rats (Fig. 2B).

## MVP functions as a cell survival factor

**Figure 3:**
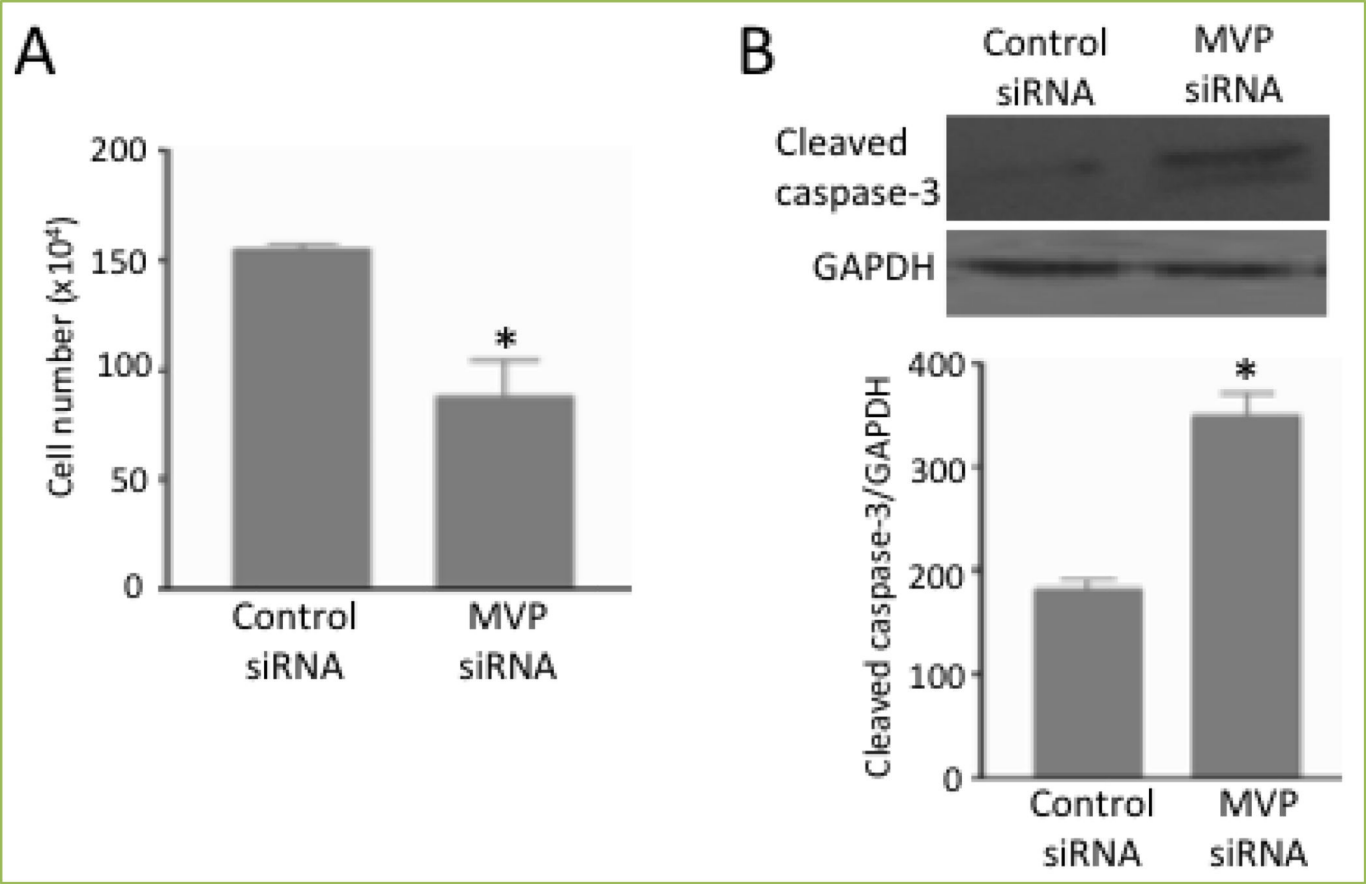
MVP protects against cell death Human bronchial smooth muscle cells were transfected with control siRNA or siRNA to knockdown MVP. (A) The cell number was determined by counting on a hemocytometer. (B) Cell lysates were subjected to immunoblotting to monitor cleaved caspase-3 expression. Bar graphs represent means ± SEM. * denotes values significantly different from the control at *P*<0.05. Reprinted with permission [2].

One function of MVP in smooth muscle appears to involve defining the fate of cells. As shown in Fig. 3A, the siRNA knockdown of MVP reduced the number of human airway smooth muscle cells^[2]^. Fig. 3B shows that this was accompanied by the formation of cleaved caspase 3, a marker of apoptosis^[2]^. Thus, MVP appears to serve as a cell survival factor. Moreover, the MVP regulation of heat-induced cell death suggests the existence of MVP actions that are distinct from the drug sequestration^[2]^.

**Figure 4:**
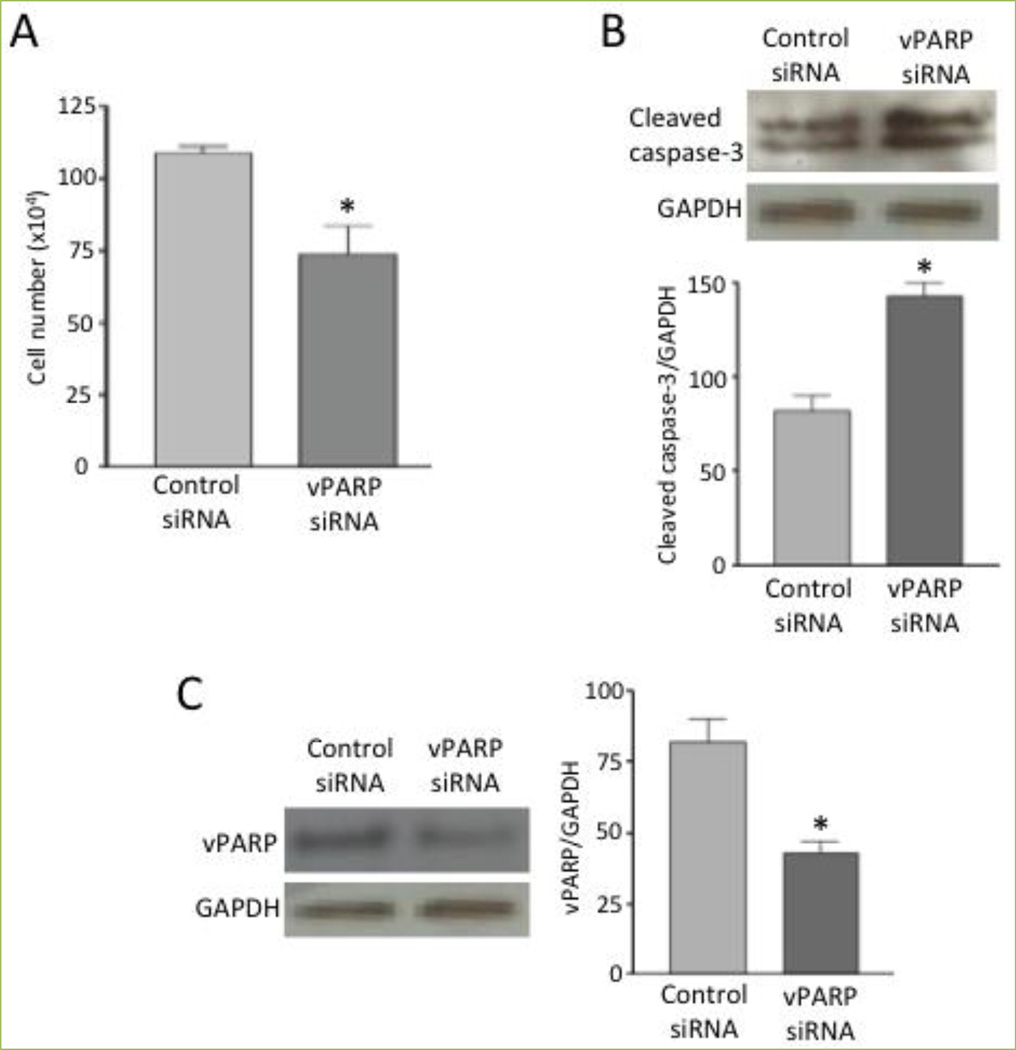
vPARP protects against cell death Human bronchial smooth muscle cells were transfected with control siRNA or siRNA to knockdown vPARP (Santa Cruz). (A) The cell number was determined by counting on a hemocytometer. (B & C) Cell lysates were subjected to immunoblotting to monitor the expression of cleaved caspase-3 (Cell Signaling Technology, Danvers, MA, USA) and vPARP (Santa Cruz). Bar graphs represent means ± SEM (n = 4). * denotes values significantly different from control at *P*<0.05.

The cell survival role of MVP is likely due to its function as a part of the vault, as vPARP -- another component of the vault -- also serves a similar role. As shown in Fig. 4A, the knockdown of vPARP in human airway smooth muscle cells by siRNA caused a significant reduction in cell number. vPARP knockdown also promoted apoptosis as monitored by measuring caspase-3 cleavage (Fig. 4B). Fig. 4C shows the extent of vPARP knockdown by siRNA. These results suggest that both MVP and vPARP are needed for cell survival, strengthening the notion that these proteins act as part of the vault particle.

## Post-translational modifications of MVP (S-glutathionylation)

**Figure 5:**
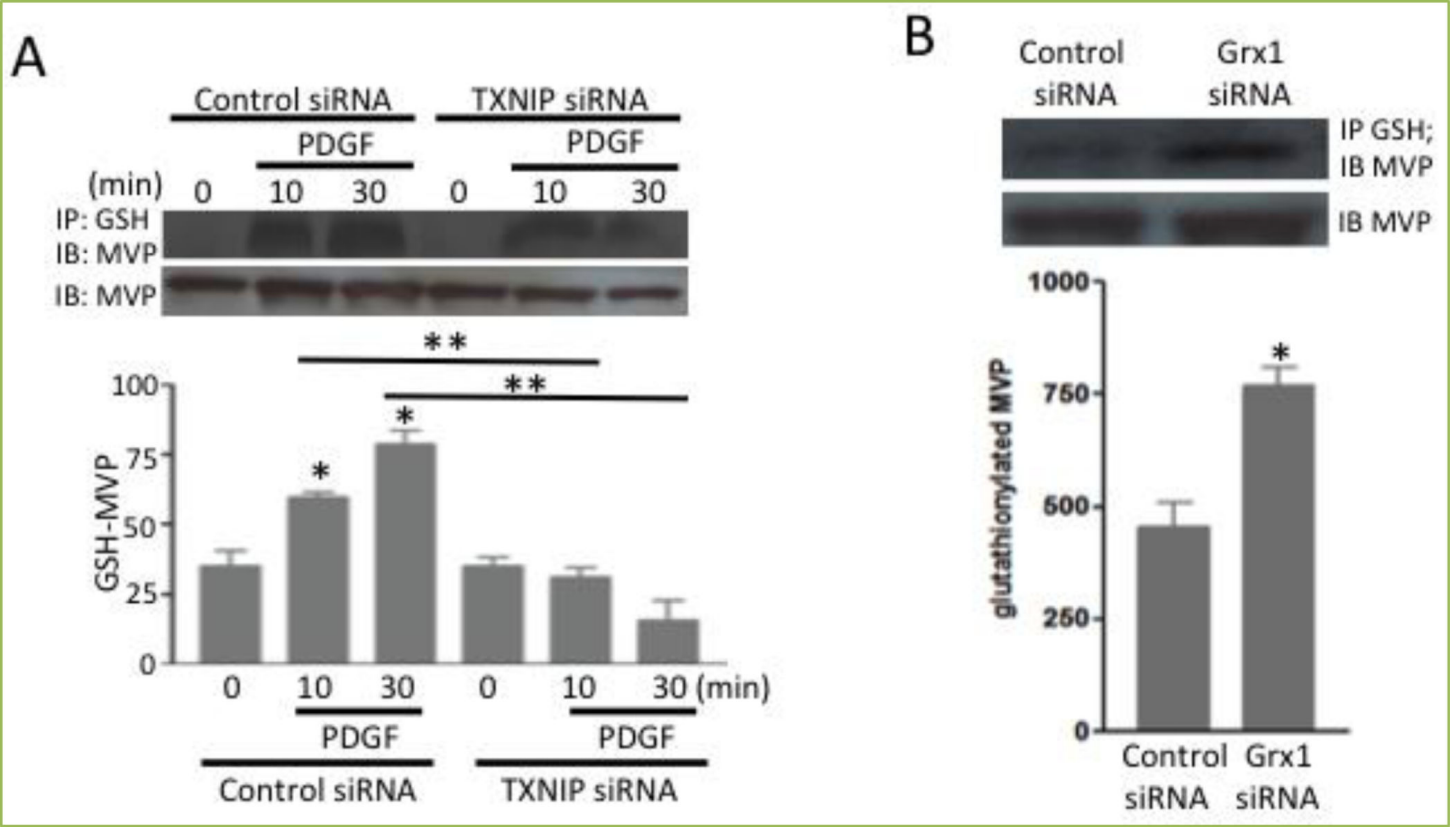
MVP is glutathionylated (A) Human bronchial smooth muscle cells were transfected with thioredoxin-interacting protein (TXNIP) siRNA and treated with platelet-derived growth factor (PDGF). (B) Cells were transfected with siRNA to knockdown glutaredoxin 1 (Grx1). Cell lysates were immunoprecipitated with the glutathione (GSH) antibody, followed by immunoblotting with the MVP antibody. Bar graphs represent means ± SEM. * denotes values significantly different from the control, and ** denotes values significantly different from each other at *P*<0.05. Reprinted with permission [2].

Recently, our laboratory reported that the treatment of HBSMCs with growth factors such as PDGF caused post-translational modifications of proteins via S-glutathionylation, a process of adding glutathione to cysteine residues^[2]^. Mass spectrometry revealed that MVP is the protein that is S-glutathionylated. Immunoprecipitation with the glutathione antibody followed by non-reducing SDS-PAGE and immunoblotting with the MVP antibody confirmed the mass spectrometry results by showing that PDGF promoted the S-glutathionylation of MVP after 10 min of cell stimulation (Fig. 5A). The S-glutathionylation of MVP was also promoted by knocking down glutaredoxin 1, a protein that catalyzes the deglutathionylation reaction (Fig. 5B). Conversely, the siRNA knockdown of thioredoxin-interacting protein, which is an endogenous inhibitor of thioredoxin, inhibited platelet-PDGF-induced MVP glutathionylation (Fig. 5A).

**Figure 6:**
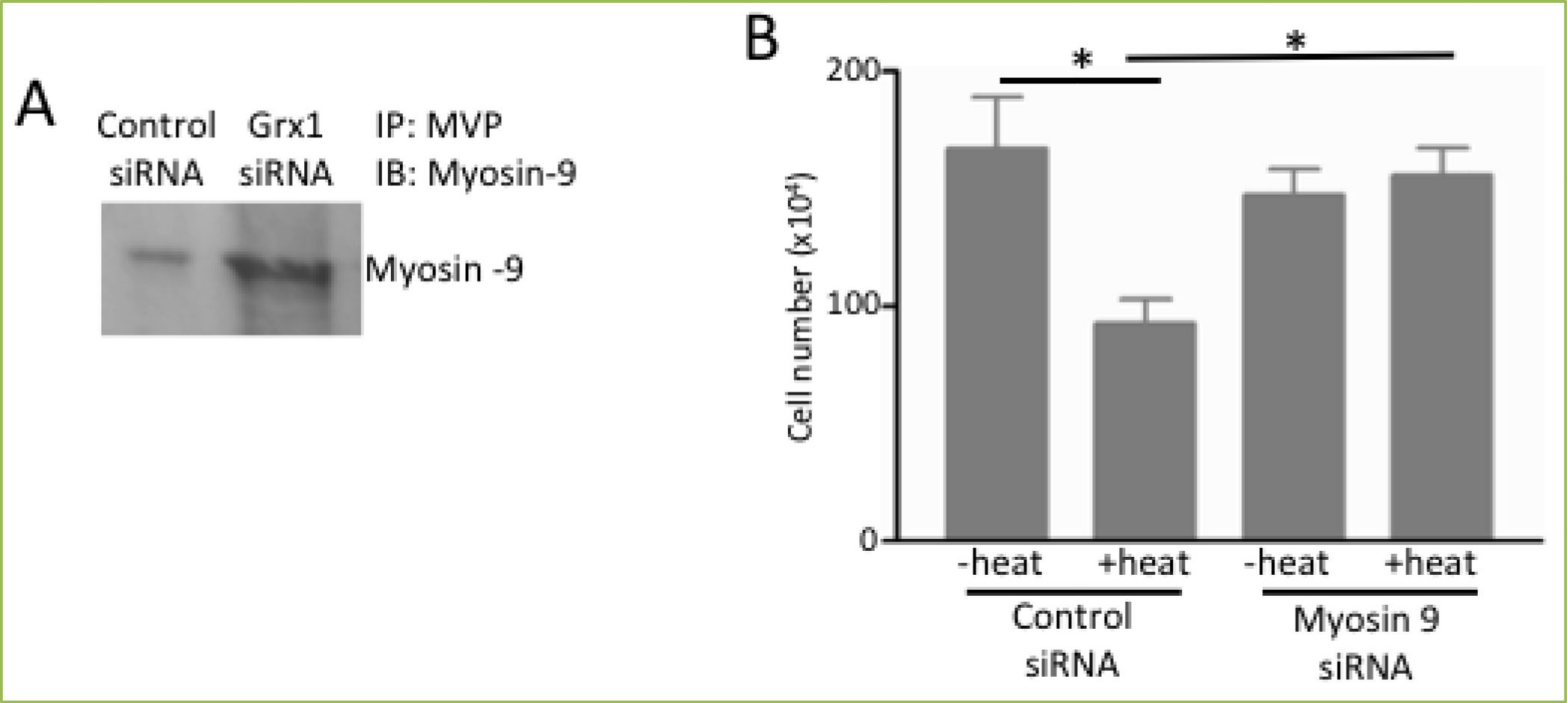
MVP interacts with MYH9 to mediate cell death in a glutaredoxin 1-dependent manner (A) Human bronchial smooth muscle cells were transfected with control or Grx1 siRNA. Cell lysates were immunoprecipitated with the MVP antibody and subjected to immunoblotting with the myosin 9 antibody. (B) Human bronchial SMCs transfected with control siRNA or siRNA to knockdown myosin 9 siRNA. Cells were then heated at 65°C for 10 sec and incubated at 37°C for 2 h. The cell number was determined by counting on a hemocytometer. The bar graph represents means ± SEM. * denotes values significantly different from each other at *P*<0.05. Reprinted with permission [2].

To identify the function of the redox regulation of MVP, we tested the hypothesis that protein-protein interactions with MVP may occur in a protein S-glutathionylation-regulated fashion. This hypothesis was tested by treating HBSMCs with control siRNA or siRNA for glutaredoxin 1, immunoprecipitating cell lysates with MVP, running SDS-PAGE and staining proteins in the gel with Coomassie Blue. Through this approach, we identified a protein in samples from cells in which glutaredoxin 1 was knocked down (thereby increasing protein S-glutathionylation), but not from cells treated with control siRNA. Mass spectrometry identified that this protein is non-muscle myosin heavy chain 9 (MYH9), and this finding was confirmed by immunoprecipitation/Western blotting (Fig. 6A). The siRNA knockdown of MYH9 inhibited cell death (Fig. 6B), suggesting that MYH9 serves as a mediator of cell death^[2]^.

**Figure 7:**
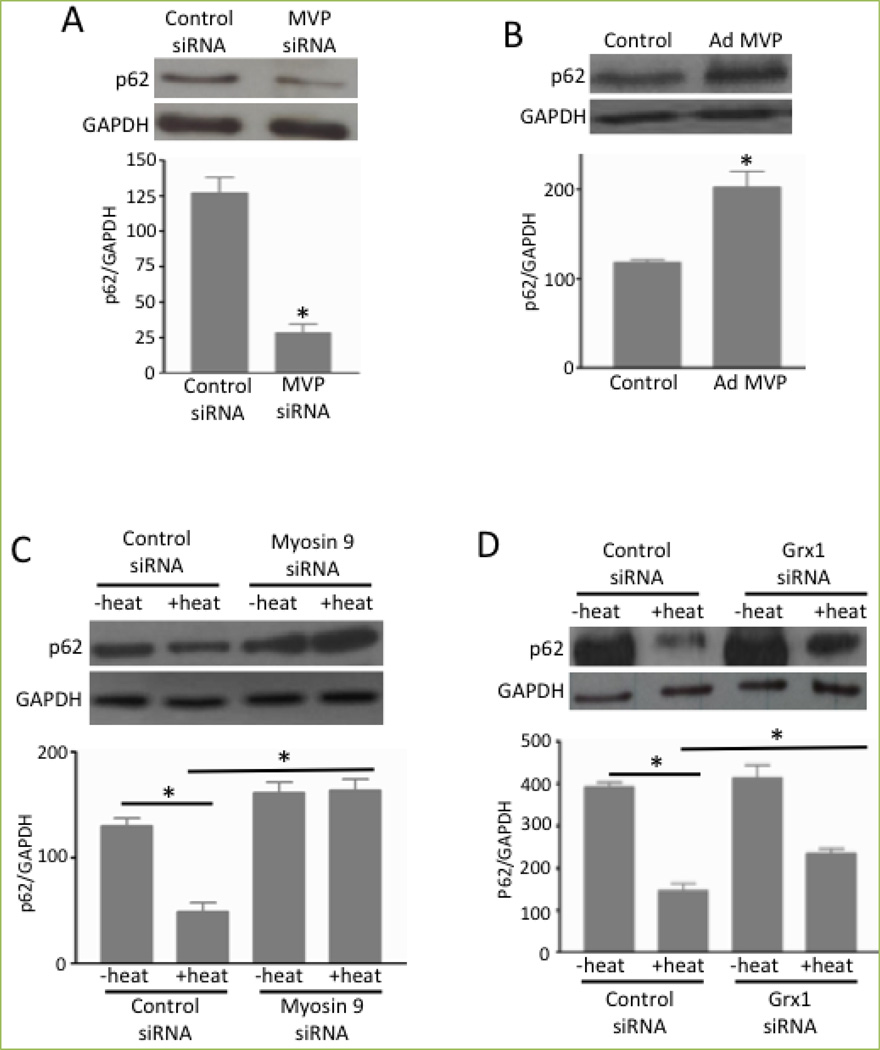
MVP and MYH9 regulate autophagy (A & B) Human bronchial smooth muscle cells were transfected with siRNA to knockdown MVP or treated with adenovirus (Ad) to overexpress MVP. Cell lysates were subjected to immunoblotting to monitor p62 expression. Bar graphs represent means ± SEM. * denotes values significantly different from the control at *P*<0.05. (C & D) Human bronchial smooth muscle cells transfected with siRNA to knockdown myosin 9 siRNA or to knockdown Grx1. Cells were then heated at 65°C for 10 sec and incubated at 37°C for 2 h. Cell lysates were subjected to immunoblotting to monitor the expression of p62. Bar graphs represent means ± SEM. * denotes values significantly different from each other at *P*<0.05. Reprinted with permission [2].

**Figure 8:**
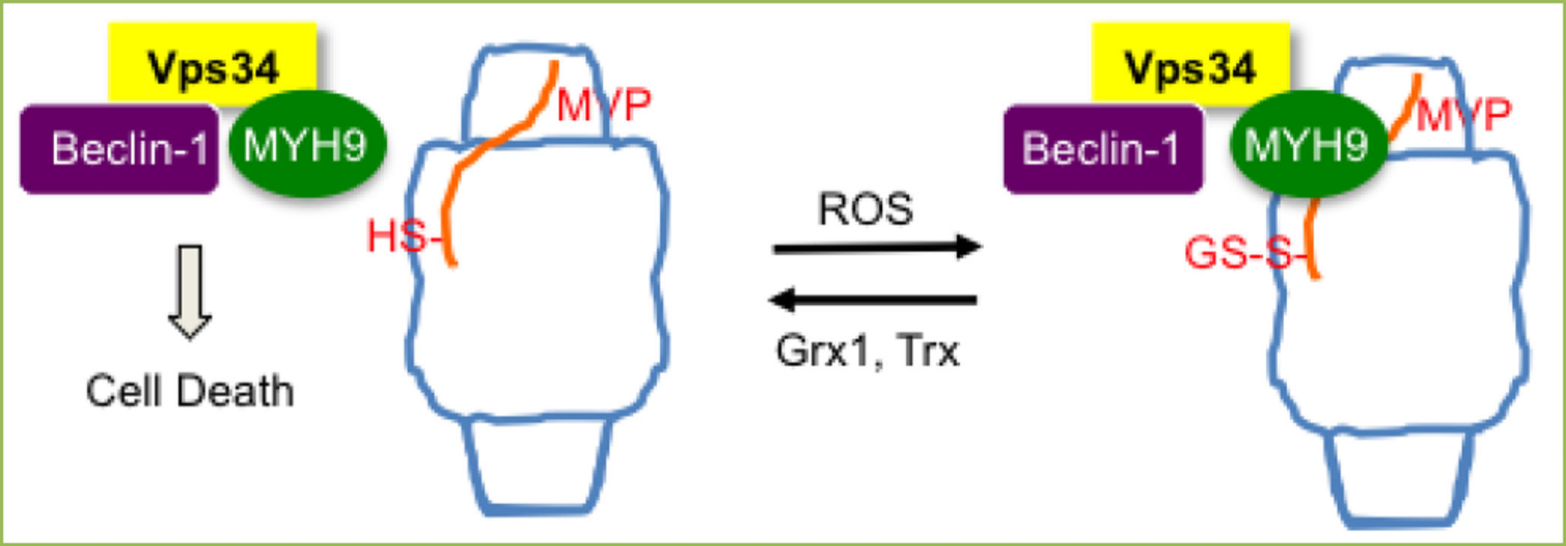
A proposed mechanism for the role of MVP in Beclin-1/Vps34/MYH9 complex-mediated cell death The ROS-dependent *S*-glutathionylation of MVP triggers the binding of the MYH9 complex along with vascular protein sorting 34 (Vps34) and Beclin-1 to the vault complex, inhibiting Beclin-1-dependent cell death. Glutaredoxin 1 (Grx1) and thioredoxin (Trx), which inhibit this cell survival mechanism, enhance smooth muscle cell death.

A series of experimental observations suggested that the deglutathionylation of MVP and thus the dissociation of MYH9 from MVP promote autophagy-mediated cell death. MVP knockdown caused the downregulation of p62 (Fig. 7A), indicating the activation of autophagy, while MVP overexpression had the opposite effect (Fig. 7B). On the other hand, the heat-induced downregulation of p62 was completely inhibited by MYH9 knockdown (Fig. 7C) and was partially but significantly inhibited by glutaredoxin 1 knockdown (Fig. 7D). Since MYH9 has been shown to interact with Vps34^[18]^, which binds to Beclin-1 for the activation of autophagy^[3]^, we propose that Beclin-1, Vps34 and MYH9 form a complex that mediates cell death and that the ROS-dependent S-glutathionylation of MVP triggers the binding of MYH9 to the vault complex, inhibiting Beclin-1-dependent cell death (Fig. 8).

## Post-translational modifications of MVP (ubiquitination)

To determine the efficacy of a clinically used proteasome inhibitor on reversing pulmonary vascular remodeling by killing PA smooth muscle cells, we treated rats with PAH as well as proliferating cultured HPASMCs with carfilzomib. We found that carfilzomib effectively killed human PA smooth muscle cells and reversed pulmonary vascular remodeling in intact rats^[17]^. We further found that ubiquitin is essential for carfilzomib-induced cell death and some proteins are ubiquitinated in response to carfilzomib treatment. Mass spectrometry identified that one of the proteins ubiquitinated by carfilzomib is MVP, and this was confirmed by immunopecipitation and immunoblotting^[17]^. The ubiquitination of MVP is promoted in PAs in response to treating rats with PAH by carfilzomib, but not in healthy control rats^[17]^. This ubiquitination of MVP in the remodeled pulmonary vasculature with thickened PA smooth muscle was observed in two different rat models of PAH, namely SU5416 injection plus hypoxia and SU5416 injection plus ovalbumin immunization without changing the expression levels of MVP^[17]^.

## Summary

MVP, the major constituent of vault particles is expressed in cardiac and smooth muscle and may play important roles in cardiovascular pathophysiology. It appears that one function of MVP in smooth muscle includes being a cell survival factor. Such a mechanism may include the regulation of autophagic cell death through the interactions of MVP with Beclin-1 and its associated proteins. In smooth muscle cells, MVP can be S-glutathionylated or ubiquitinated. These post-translational modification mechanisms of MVP may be involved in cell regulatory processes. Investigating the functions and regulatory mechanisms of MVP and the vault particle in cardiac and smooth muscle presents an exciting new research area in cardiovascular/pulmonary biology.

## Abbreviations

HBSMCs: human bronchial smooth muscle cells
HPASMCs: human pulmonary artery smooth muscle cells
LV: left ventricle
MVP: major vault protein
MYH9: non-muscle myosin heavy chain 9
PA: pulmonary artery
PAH: pulmonary arterial hypertension
PDGF: platelet-derived growth factor
RV: right ventricle
SMCs: smooth muscle cells
TEP1: telomerase-associated protein-1
vPARP: vault poly(ADP-ribose) polymerase
